# Addressing Structural Racism Using a Whole-Scale Planning Process in a Single Academic Center

**DOI:** 10.1089/heq.2023.0093

**Published:** 2023-09-13

**Authors:** Eve J. Higginbotham, Kya Hertz, Corrinne Fahl, Dwaine B. Duckett, Kevin Mahoney, J. Larry Jameson

**Affiliations:** Perelman School of Medicine, Office of the Dean, Department of Ophthalmology, University of Pennsylvania, Philadelphia, Pennsylvania, USA.

**Keywords:** structural racism, change management, inclusive culture, health equity, strategic planning

## Abstract

**Purpose:**

The murder of George Floyd in 2020 prompted a national demand for cultural transformation to confront the systemic racism prevalent in the country. Academic medical centers were not exempt from this urgent call. This article evaluates the efficacy of a strategic process in fostering cultural transformation within an academic medical system.

**Methods:**

A whole-scale strategic planning process was implemented over 13 months, involving multiple working groups representing key stakeholders from each entity across the system, an anonymous survey, a communication plan, and a balanced scorecard to monitor progress. More than 5500 voices, 160 recommendations, 122 data gathering sessions, and town hall meetings contributed to the creation and implementation of vital action items and a strategic framework. The Diversity Engagement Survey (DES) was administered 18 months following the process launch.

**Results:**

Of the 45,554 employees, students, faculty, and trainees, 96.5% completed unconscious bias education within the fiscal year and 76% of action items, termed “Just Do Its,” were completed. Mission, vision, values, and strategic priorities were crafted to serve as a framework for intermediate and long-term actions. The DES revealed improvement in the “respect” attribute of an inclusive culture, and 64% of respondents confirmed that action for cultural transformation is addressing racism both within and outside of the institution.

**Conclusion:**

Implementing a shared purpose, engaging multiple working groups representing key stakeholders, and empowerment of stakeholders to implement changes, in conjunction with the development of a strategic framework addressing structural racism, resulted in the completion of vital action items to initiate cultural change.

## Introduction

During the pandemic that began in 2020, the national conversation shifted in sharp focus to the structural inequities that have shaped our society for centuries. Although the disproportionate impact of the pandemic on communities of color^1^ had already garnered the attention of leaders across the spectrum of health care, private corporations, federal agencies, and congressional leadership, it was the murder of George Floyd in May of 2020^2^ that struck the heart and soul of the nation. The confluence of the nation's undivided attention, inequities observed during the pandemic, and the continued evidence of inequities in interactions between police and the black and brown communities created a national reckoning regarding structural racism not only across the country, but within organizations, including academic medical centers.

These impactors also affected the health care industry, especially those organizations responsible for the health of populations. Health care organizations cannot attain health equity unless there is a collective dedication to this goal, which is reaffirmed daily through an inclusive culture.

Strategic planning can be an effective tool to assist an organization moving in a new direction. It is an important exercise used to set goals, develop strategies, identify action items, all guided by mission and vision statements.^3,4^ Often these planning exercises are preceded by an analysis of the internal and external environments, with an assessment of the strengths, weaknesses, opportunities, and threats impacting the organization's current state and its future. Given the urgency presented by the cultural influences that surfaced in early 2020, a different model for planning was needed to address the diverse interests of organizational stakeholders and the urgency to acknowledge and address long-standing, institutional contributors to structural racism. This case study demonstrates the utility of adapting elements of whole-scale change (WSC),^5^ applying this methodology across a health care system using a virtual platform to initiate cultural change.

There are several methods to achieve cultural change in organizations ^6^; whole-scale strategic planning^7^ uniquely provides the opportunity to harness the energy of diverse stakeholders across an organization, specifically in this case, to address the effects of structural racism. Previous reports of the application of WSC^5^ have focused on the strategic alignment between a hospital and schools of medicine, nursing, pharmacy, and dentistry at a single institution;^6^ and the professional development and advancement of nurses in a hospital system.^8^ To our knowledge, this is the first report of an academic medical center that used WSC^5^ to advance efforts to address structural racism.

WSC^5^ is an appropriate method, given the pressing need to involve diverse perspectives in a complex health system during a period of ambiguity. An uncertain landscape necessitates a compelling common purpose to anchor the efforts and involve a broad cross section of the organization. Dannemiller and coauthors^5^ noted guiding WSC principles, including the establishment of a common purpose, the involvement of working groups comprising representative stakeholders, the implementation of strategies to engage and empower the entire organization, and the continuous evaluation of outcomes to inform future actions. A salient feature of this process is the engagement of the “microcosm of the whole.”

## Methods: Context and Process Overview

Penn Medicine is an integrated health system including a school of medicine and six hospitals. The corporate leadership structure includes the Chief Executive Officer (CEO) of the University of Pennsylvania Health System (UPHS) reporting to the Dean of the Perelman School of Medicine, and who also serves as the Executive Vice President of UPHS. This position reports to the President of the University. The governance of Penn Medicine is shared between the Penn Medicine Board and the University of Pennsylvania Board of Trustees.

The UPHS patient care facilities include the following: the Hospital of the University of Pennsylvania and Penn Presbyterian Medical Center, Chester County Hospital; Lancaster General Health, Penn Medicine Princeton Health, and Pennsylvania Hospital. Additional facilities and enterprises include Good Shepherd Penn Partners, Penn Medicine at Home, Lancaster Behavioral Health Hospital, and Princeton House Behavioral Health. The Perelman School of Medicine and the UPHS comprise Penn Medicine, constituting a community of 45,554 faculty, staff, students, and trainees.

The planning process described here spans 13 months, July 1, 2020–August 31, 2021. Following the murder of George Floyd on May 25, 2020, the Office of Inclusion, Diversity, and Equity (OIDE) requested solidarity statements from across Penn Medicine. To begin this process, two town hall meetings were hosted to initiate a community-wide discussion regarding structural racism. The first virtual meeting was facilitated by two health system leaders. A second virtual town hall meeting was hosted by the Dean, CEO of UPHS, and the CEO of Children's Hospital of Philadelphia, which also employs members of the university faculty.

The number of attendees in both combined was 2150, among the highest attendance of any previous virtual meetings. The second meeting included a panel that consisted of senior and junior faculty, students, staff, and a postdoctoral fellow, who were questioned regarding their reflections and experiences related to structural racism and their hope for the future. Comments and recommendations were electronically submitted by participants throughout this meeting. Additional input was also submitted either formally to executive leadership or directly to the website of the OIDE. Six themes emerged from the comments offered throughout this initial process: Culture, People, Community, Clinical, Education, and Research. These six areas served as the backbone for the strategic framework.

The OIDE of Penn Medicine was designated as the primary recipient of recommendations and coordinator of the strategy to respond to the Penn Medicine community, providing a pivotal role and leveraging their expertise in advancing inclusion, diversity, and equity. To manage the high demands related to the hospital system, a formal partnership was established with the Senior Vice President from Human Resources of UPHS. In addition, a core group of key stakeholders representing interests in the six priorities was assembled. This group met weekly, requesting additional input from the community related to recommendations, and establishing a mechanism to allow “first steps” to be implemented as the plan was being developed. To address the strong interest in prompt action, immediate action items were identified as “Just Do Its” (JDIs), which were considered both overdue and achievable within 6 to 9 months.

Within the first 6 months of launching the formal process, data gathering sessions^9^ involving 12% of the entire workforce, students, and trainees were facilitated. From August through November 2020, a total of 170 volunteers conducted 122 sessions. The session facilitators were volunteers, department chairs, and individuals identified by their manager. A facilitator and notetaker were assigned to each session. Anonymity was emphasized to create a safe space for participants to candidly discuss topics. Facilitators were provided with a guide, presentation, scheduling template, note capture template, and a set of questions to ensure consistency across all sessions. In addition, participants were offered the option to anonymously respond to the questions electronically. More than 5500 responses were collected from the initial town hall and data gathering sessions.

After concluding all sessions, the data were then categorized into six priority areas identified during the initial town hall and presented at a retreat attended by more than 60 stakeholders from across the organization, who worked collaboratively to refine the vision, mission, and values and identify strategic priorities and tactics. The retreat comprised six breakout sessions, each focusing on a specific strategic framework area: Culture, People, Community, Clinical, Education, and Research.

Next, an executive leadership-endorsed governance structure was created. Executive leadership and this governance structure reviewed and approved the final strategic roadmap, including mission, vision, values, strategic priorities, and supporting tactics. Nine months into the work, the strategic roadmap was presented to the Penn Medicine Board, along with a balanced scorecard (BSC)^9–11^ with the purpose of evaluating the program and tracking progress. This tool balances leading indicators such as infrastructure and key processes with lagging indicators, specifically, stakeholder satisfaction and financial stewardship, ensuring sustainability of the plan.^12,13^

The BSC listing of JDIs was made available online during the initial 6 months. This affirmed transparency of the process, documented identified areas of commitment, and monitored performance metrics. The evaluation of this program is based on the progress made on these actions.

Between months 6 and 8, individuals from across Penn Medicine were nominated to colead each strategic priority area, that is, Culture, People, Community, Clinical, Education, and Research. These coleaders, along with the core planning team, selected 11 actions to launch the strategic plan. These action items were shared with governance members and presented to executive leadership. Individuals representing stakeholders from across Penn Medicine were assigned to execute each tactic. After 8 months the strategic plan was launched.

An early communication plan was developed, consisting of weekly updates, posting of the BSC to the OIDE website, and periodic presentations to executive leadership, governance bodies, and human resources leadership. After the 1st year of implementation, a town hall meeting was held across the institution to report on the progress made.

### Assessment of the effectiveness of the strategic framework

Eighteen months following the launch of the strategic framework and the completion of the 11 strategic action items, the Diversity Engagement Survey (DES)^14^ was administered and the results were compared with previous use of the DES in 2015 and 2018. The University of Pennsylvania's Institutional Review Board approved the use of the DES. The recommendations outlined in the 11 strategic initiatives were advanced to executive leadership for approval at 24 months.

## Results

### Expression of shared dissatisfaction with the *status quo*

A key component of this process was the acknowledged dissatisfaction of the *status quo* by many leaders, faculty, staff, trainees, and students. This dissatisfaction was expressed through solidarity statements, which were posted on the OIDE website and shared with the broader community. Thirteen solidarity statements were collected; contributors to these statements included the dean, decanal staff, department chairs, unit leaders, and the CEO of the hospital system.

### Establishing and communicating a model for change: action for cultural transformation

**FIG. 1.**
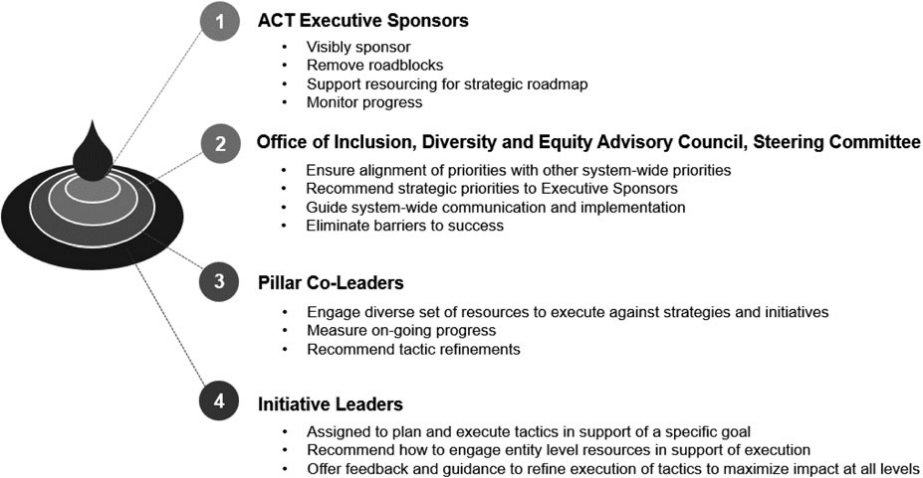
Governance of action for cultural transformation. ACT, action for cultural transformation.

**FIG. 2.**
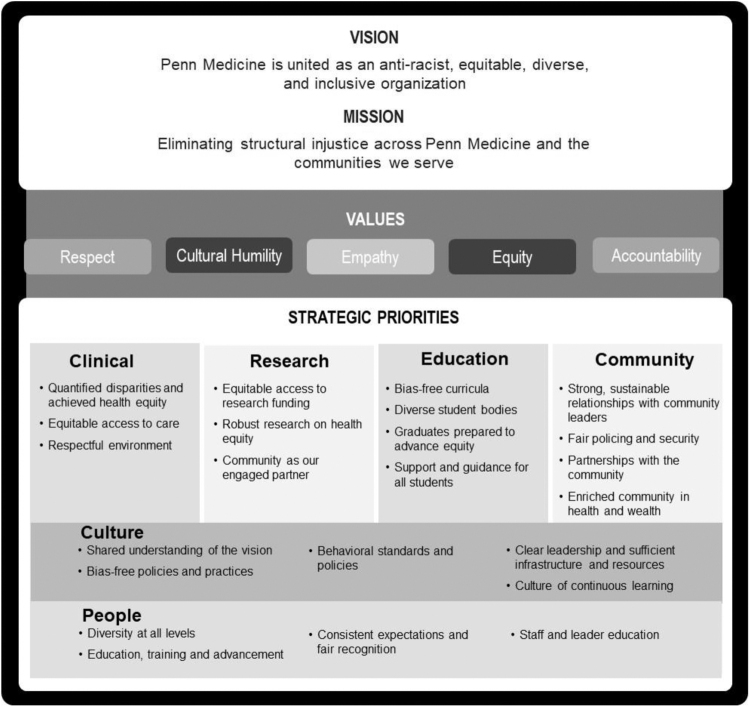
Mission, vision, values, and strategic priorities of ACT, including the six areas of focus: clinical, research, education, community, culture, and people.

Based on the energy generated by the town hall meetings, a statement of commitment to address systemic inequities was shared. Leadership was conscious of the need to be action oriented. The organization's statement of commitment was articulated as action for cultural transformation (ACT) with a specific initial action for all staff, faculty, students, and trainees: mandatory unconscious bias education. By the end of the fiscal year, 96.5% of the 45,554 members of the community completed either a 1-h virtual training or an on-demand training session. The governance model for ACT (Fig. 1) and the strategic roadmap were developed concurrently (Fig. 2).

#### JDIs and the creation of the balanced scorecard

JDIs included those items that could be accomplished in 6 to 9 months, as the strategic plan developed. By the end of the fiscal year, 54 (76%) of the JDIs were completed. The list of initial JDIs was reviewed at 13 months for specificity, clear metrics, and time line. Eight of the original 76 JDIs submitted were removed, and three were added, resulting in 71 that were evaluated. The distribution of JDIs based on BSC domains was as follows: 30.9% Organizational Infrastructure, 42.2% Internal Process, 12.7% Financial Stewardship, and 14.1% Stakeholder Satisfaction. By ACT Pillar: 2.8% Clinical, 1.4% Community, 22.5% Culture, 50.7% Education, 19.7% People, and 2.8% Research. Table 1 lists representative action items.

**Table 1.** Selected “Just Do Its” in the Balanced Scorecard Format

Action	ACT category	Status	Metrics
Organizational infrastructure			
Unconscious Bias Training—Leaders—3 months; all Penn Medicine. Employees (OIDE)	Education	Complete	Confirm training for all leaders and employees
Dermatology Diversity and Community Engagement Residency training position	People	Complete	Resident selected
Internal processes			
Identify specialty-specific disparities in health outcomes and access to care to as quality goals for the department and Penn Medicine (CPUP)	Clinical	Complete	Improve health outcomes and access to care for our local community
Restructure PSOM curriculum and operations to emphasize antiracist education (Senior Vice Dean for Medical Education)	Education	Complete	The next phase of curriculum review is to develop Medical Education Program Objectives. The use of current CDC categories for race and ethnicity are preferred. Preclerkship curriculum:
Financial stewardship			
Establish grants for health disparities research and/or coursework (Senior Vice Dean for Medical Education)	Education	Complete	Grants have been identified and internal funding established. No applications were received for FY21, and the process will be reevaluated for FY22 to be more encouraging for student applications
Work with Development to increase available funding for recruitment and retention of diverse faculty and students (OIDE/Development)^*^	People	Complete (key milestone)	Increased available funds for all tracks as well as bridge funding
Stakeholder satisfaction			
Assert common purpose related to commitment to address structural racism at Penn Medicine, collect statement from departments, Health System Units, Centers, and Institutes (OIDE)	Culture	Complete	Statements posted to OIDE website
Create opportunities to listen to student concerns, experiences, and suggestions for change	Education	Complete	Student feedback

^*^ Office of Inclusion, Diversity, and Equity and Development.

ACT, action for cultural transformation; CDC, Center for Disease Control; CPUP, Clinical Practices of the University of Pennsylvania; FY21, Fiscal year 21; FY22, Fiscal year 22; OIDE, Office of Inclusion, Diversity, and Equity; PSOM, Perelman School of Medicine.

#### Implementation of the ACT strategic plan

**FIG. 3.**
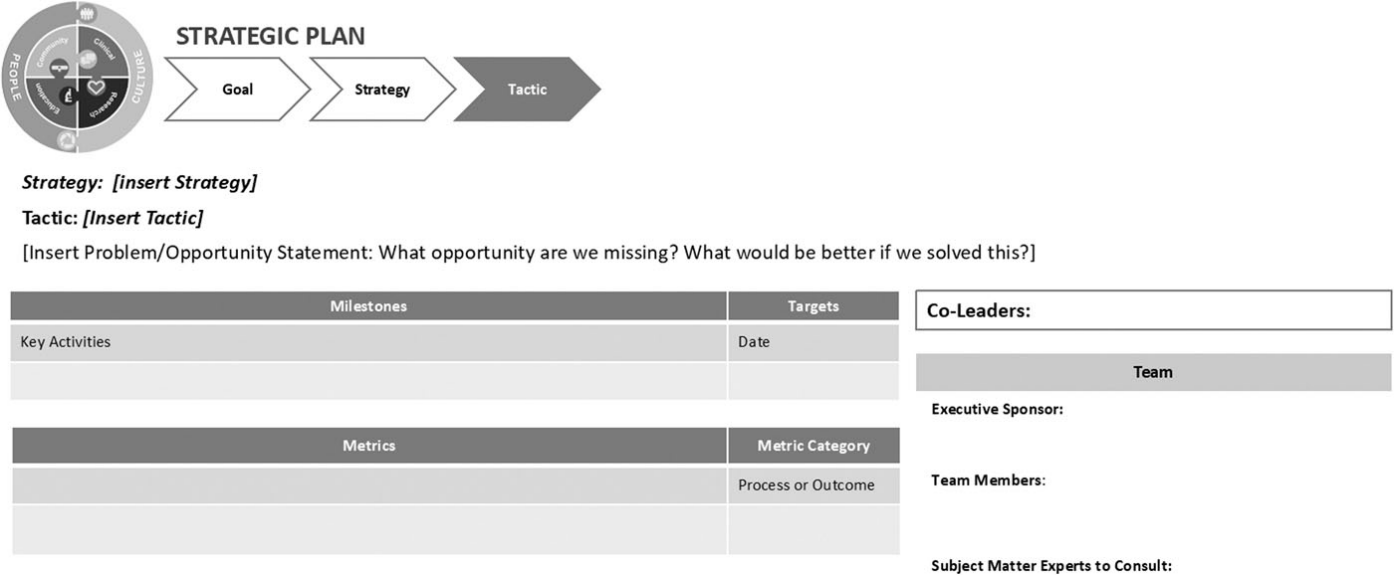
Example template used by Pillar and Initiative Leaders to develop their strategic plan.

A charter process ensured that each group responsible for executing a tactic enlisted the support of appropriate team members, remained goal oriented, and tracked the progress against key milestones and metrics. Figure 3 represents the model for the charter that guided each tactic. By month 24, all 11 teams advanced recommendations to the executive leadership for approval and implementation. Table 2 contains a summary of selected recommendations and outcomes.

**Table 2.** Action for Cultural Transformation Strategic Initiative Overview

Pillar	ACT initiative	Recommendation	Key actions
Culture	Vice Chair, IDE, committee Support initiative	Inventory of health system IDE councils and Vice Chairs Establish annual IDE Summit	Established a more cohesive network of IDE committees, chairs, and individuals across Penn Medicine to effectively amplify initiatives Launched inaugural ACT/IDE summit in September 2023
Culture	Affinity groups Initiative	Create Penn Medicine Community Groups	Initiated a supportive employee resource group to cultivate a culture of inclusion and diversity for UPHS
Culture	Designing forums Initiative	Revise training and toolkits for facilitating conversations	Designed forums at multiple levels of the organization to ensure employees and leaders are engaged, learning, appraised of progress, and deepening the collective understanding of inclusion, diversity, and equity
Culture	Communications Initiative	Enhance centralized communications. Increase transparency and institutional humility in content	Implemented text-based communication, subscription, and centralized communication hub to alleviate the racialized hierarchy-based digital divide within our system
Community	Community infrastructure Initiative	Approve funding to create Center for Community engagement	Established a community infrastructure to concentrate on enhancing services to underserved communities:
Clinical	Health equity improvement Program initiative	Create health equity improvement Collaborative Training Program Progress on two health equity goals; increase colon screenings and reduce major maternal morbidity and mortality	Expanded a health equity course focused on collaborative learning with the goal of improving equity problems Increased colon screenings by 70+% Reduced major maternal morbidity and mortality by 29+%
Clinical	Reporting and addressing discriminatory behavior	Create a quarterly bias reporting	Created a reporting structure to mitigate discriminatory behavior, colleague to colleague, patient to provider, and provider to patient Piloted an intervention process for those individuals and/or units where there are patterns of discriminatory/biased behavior
People	Employee relations Initiative	System-wide reporting forum, entry-level reporting huddles	HR policies were reviewed with a focus on policy language that impacts diverse employee populations Analysis of HR policies informed ongoing manager education on fair and equitable execution of policies and procedures and equitable opportunities
People	Employee hiring, professional development, and mobility initiative	System dashboard with diversity data, standardize hiring processes, create diversity career resource center	Created a system dashboard with appropriate diversity data to support a culture of accountability and the recognition of progress Introduced a “reverse mentoring” program, where community health workers educate senior UPHS Leaders on issues around community connection, health care access, and employment opportunity Established Penn Medicine and Pathways Emerging Careers program, and the internal Pathways to Promotion program, to provide employment and mobility opportunities to hundreds of employees and members of the community
Education	Student Advocacy and Support Initiative	Approved funding to launch IDEAL research program	Support system for medical and graduate students underwent structural reorganization Critical changes to the UME curriculum to address language that may be deemed as biased
Research	Cluster hiring Initiative	Hire faculty in clinical and basic science clusters with focus on health equity and IDE	Increase in the number of presidential professors for diverse faculty: 5 new diverse faculty hired

HR, Human Resources; IDE, Inclusion, Diversity, and Equity; IDEAL, Inclusion, Diversity, Equity, and Learner; UME, Undergraduate Medical Education; UPHS, University of Pennsylvania Health System.

#### Diversity Engagement Survey

The DES,^14^ which had been administered in 2015 and 2018, was compared with the findings of 2021. The 2021 results showed improvement in “respect.” Notably, there was also an increase in the participation, measuring 10,468 total respondents, which was 95% greater than 2018 and representing 23% of potential respondents. This iteration contained a new custom question “I feel that action for cultural transformation (ACT) is initiating change in my institution.” A total of 8928 respondents answered this question; 64% agreed with the statement and 31% were neutral.

## Discussion: Lessons Learned

The WSC process^5^ was critical to the launch of this strategic plan to address structural racism in the academic medical center. The pain, disappointment, frustration, anger, and sadness that fueled the protests in spring and summer 2020 were channeled into a collective process that translated into constructive actions. The benefits of this approach have been previously highlighted compared with a more traditional approach to planning, which involves typically a small leadership team and a top-down model.^7^ Moreover, the inclusive engagement of all members of the organization is essential and the use of a virtual platform may have facilitated that engagement. In addition to engaging as many staff, students, trainees, faculty, and administrators as possible, it is important to have leadership respond voluntarily to ensure trustworthiness and transparency of the planning process at every stage.^15^

Moreover, this WSC approach captures the wisdom of those individuals operating closer to the front lines while remaining guided by a structured process to facilitate alignment. Bringing together a critical mass of individuals motivated to work toward change and then coming to a consensus on the vision of the new transformed state were important launchpads, exemplified by the solidarity statements produced at the beginning of the process.

One of the guiding principles of WSC,^5^ “tapping in the microcosm of the whole” was important at every stage, beginning with the core working group, the data gathering sessions, the retreat, components of the governance structure such as the steering committee and pillar coleads, and the current working groups focused on the action items. These groups included a diversity of representative stakeholders including the school of medicine constituents and system employees in the process. The full engagement of executive leadership at every stage is essential. The driving purpose of ACT, to address racism head on, was most effectively executed using this WSC approach. It is also important to note that the ACT aligns with the Anti Racism Framework recommended by the National Association of Diversity Officers in Higher Education with action items in all of the priority areas outlined in the model.^16^

A primary outcome measure of this effort is the DES, a tool that has been validated to assess an inclusive culture.^14^ Two previous DES assessments serve as the baseline for measuring cultural change in the future and were critical to demonstrating that change had occurred because of this process. The 95% increase in the response rate and the positive response to the question regarding ACT are indicators that the key stakeholders were positive regarding the progress made in cultural changes.

Two additional tools helpful to this process were the charter template used to launch action items and the BSC. Building diverse teams is at the core of this process and thus the charter system provided a blueprint for new leaders to bring their teams together to accomplish a common goal and structured the inclusivity of this process. Since not everyone is familiar with strategic planning, such an approach provides a way to ensure consistency. The BSC^11^ served as an important element of this process by tracking key metrics, including leading and lagging indicators for change. Given that there was minimal infrastructure and key processes in place, it is not surprising that 73.1% of the metrics tracked in the JDIs align with these two leading indicators.

It is also reassuring to have the BSC to publicly share progress with stakeholders, particularly those who remain doubtful that cultural change is possible. Moreover, accountability was a recurring theme in the data gathering sessions and became a core value of ACT. The BSC is a crucial tool to address this concern.

There are challenges launching any new initiative, particularly during a period of extreme stress on an institution on the front line of a pandemic. The strategic plan was constrained to a virtual platform. Zomorrodian^17^ noted the challenges of planning virtually, particularly related to building trust within teams and remaining proactive. There are negatives to consider when relying so heavily on this medium, since it is harder to generate side bar conversations that can drive greater innovation. The benefit of this virtual platform during this pandemic was the ability to connect many individuals in the organization. However, although there was a communication plan crafted, including electronic newsletter updates, town halls, and presentations to specific groups, there were gaps noted in the uptake of the information. Communication processes were enhanced across the entire organization to address those individuals who do not have access to electronic devices during work hours.

This strategic planning method aims to engage the entire organization in a transparent and highly interactive process, with results tracked over time. Developing a plan that addresses a human characteristic that is unconsciously affecting behavior in the workplace is not easy. It requires clear metrics, guidelines to ongoing monitoring, and provisions to account for initiative fatigue and burnout to ensure steady progress. Although institutionally there were strategic plans previously in place, developing a specific plan to advance antiracism and engage the entire organization in a transparent and highly interactive process was deemed necessary to usher in cultural change.^16^ This interactive process provided the unique opportunity for individuals to feel comfortable sharing experiences related to bias and discrimination in small groups. Continued monitoring of the progress of the strategic plan, including periodically repeating the DES,^7^ will ultimately determine its effectiveness in sustaining change.

## Conclusion

This case study demonstrates the utility and effectiveness of using a whole-scale strategic planning process to address structural racism. The key elements of this process included the active engagement of a broad cross section of the organization, frequent communication, full visible support of executive leadership, accountability, metrics, and a structural framework to support the development of core initiatives. The feedback from the organizational stakeholders demonstrated general satisfaction with the process and the outcomes.

## Abbreviations Used

ACT: action for cultural transformation
BSC: balanced scorecard
CEO: chief executive officer
DES: Diversity Engagement Survey
IDEAL: Inclusion, Diversity, Equity, and Learner
JDIs: Just Do Its
OIDE: Office of Inclusion, Diversity, and Equity
UPHS: University of Pennsylvania Health System
WSC: whole-scale change
